# Affiliative touch, sense of self and psychosis

**DOI:** 10.3389/fpsyt.2024.1497724

**Published:** 2024-11-13

**Authors:** Maurizio Peciccia

**Affiliations:** ^1^ Department of Philosophy, Social Sciences, Humanities and Education, University of Perugia, Perugia, Italy; ^2^ Gaetano Benedetti Institute of Existential Psychoanalysis, Perugia, Italy

**Keywords:** affective touch, sense of self, attachment, trauma, stress regulation, border of the self, psychosis

## Introduction

The affiliative touch represents a revolutionary innovation in the course of evolution, as it has enhanced the capacity of organisms that have adopted it to form social bonds. This is achieved through the provision of a natural mechanism for strengthening social cohesion, reducing stress and promoting mutual support within groups (1). The formation of social bonds increases the likelihood of individual survival and adaptation, and also contributes to the development of the human sense of self (2–4). The capacity to comprehend one’s own and others’ subjectivity, and to establish intersubjective bonds has facilitated social cooperation, enabling humans to modify their environment and reduce their dependence on external factors, thereby exerting a significant influence on human evolutionary development (1).

Affiliative touch has been the subject of extensive neuroscientific studies in recent decades, resulting in significant evidence concerning the neurobiological mechanisms through which it affects the formation and maintenance of attachment relationships (5, 6). These studies have also revealed its role in reducing stress experienced in social interactions (7, 8). Furthermore, its function in the development of the bodily basis for the sense of self has been clarified (9–12).

Affiliative touch, defined by slow and gentle gestures such as caresses, represents a fundamental form of interpersonal sensorimotor interaction. It plays a pivotal role in a range of evolutionary primary functions, including attachment, stress modulation, affective body representation, differentiation between self and others, the body’s ownership, and embodied simulation (4–15).

## Affective bonds and modulation of the stress axis

Affiliative touch is a pivotal factor in the formation of affective bonds and the modulation of stress. It stimulates the release of oxytocin and endorphins in the limbic system (5), which in turn increase pleasure, reduce pain, and facilitate attachment between child and caregiver (6). Furthermore, oxytocin lowers corticosteroid levels during stressful situations, thereby promoting calm and protection and strengthening attachment to the caregiver (7, 8).

Affiliative touch is most effective when performed in a repeated manner. Studies have demonstrated that oxytocin production reaches its peak after approximately half an hour of continuous, rhythmic stroking in both adults and children (14, 15).

## The bodily self and its boundaries: a multisensory affective representation

Affective touch simultaneously stimulates two distinct classes of tactile receptors: β-exteroceptors, which are connected to the somatosensory cortex, and tactile C-interoceptors, which are connected to the posterior insula (10, 11). The latter integrates information from both external and internal sources, thereby promoting a multisensory representation of the bodily self (9–12). Repeated stimulation of these receptors by affiliative touch facilitates the exchange of information between the inner and outer worlds, thereby contributing to the definition of the boundaries of the self (8, 9).

The formation of bodily self-representation is influenced by affiliative touch, which simultaneously activates the limbic system and elicits experiences of pleasure and attachment (12). This process adds an affective dimension and a sense of ownership to the bodily self-representation.

In the early stages of development, affective touch forms part of an integrated system of multisensory and motor interactions. This includes neonatal imitation (16) and affective attunement (17). These experiences contribute to a multisensory integration that supports the development of the affective representation of the bodily self (18, 19).

In affective attunement, the caregiver enhances the sensorimotor rhythm of affiliative touch by integrating touch with other sensory modalities, thereby preventing adaptation and maintaining the child’s pleasurable interest (17). The affective motivation for multisensory integration stems from the pleasure of bonding with the caregiver. The temporal rhythm of actions within the relational space acts as a unifying force and guiding principle for multisensory integration (20–22). This process supports the development of a coherent multisensory representation of the bodily self and the surrounding environment (20–22).

The affiliative touch and the motor system interact from intrauterine origins. During the fetal stage of development, the movement of both the mother and the fetus produces oscillations in the amniotic fluid, which stimulate the fetal lanugo, which is equipped with tactile C-receptors. This stimulation increases the production of oxytocin and endorphins in the limbic system, thereby generating pleasure (23). According to this well-established hypothesis, affective touch motivates fetal movement, thereby facilitating multisensory connections during the third trimester of pregnancy.

The pleasure derived from affiliative touch also influences the formation of the boundaries of the bodily self by delineating the distinction between the self and the external world (8, 9).

Federn (24), a seminal figure in psychoanalytic psychotherapy, postulated that ego boundaries are invested with affect (libido) and that ego fragility in psychotic patients is attributable to an inadequate affective investment in ego boundaries.

## The self, the self-object, and embodied simulation

The simultaneous stimulation of exteroceptive and interoceptive receptors provided by affective touch (9) confers a particular significance to the caregiver. Indeed, the caress is perceived by the receiver as both an external stimulus and as emanating from an internal organ of the body (13).

This, in addition to enhancing the effect of affective touch in the attachment process, could facilitate the formation of self-objects (25) and transitional objects (26, 27), which represent both the self and an entity separated from the self. The empirical evidence on child development indicates that the sense of self develops in two distinct but complementary ways. 1. as a discrete entity and 2. through the process of mirroring with others (28). These two modes of self-experience tend to integrate during development, resulting in a unified sense of self that is consciously perceived as separate from others while maintaining an unconscious symbiosis with them (28). From a phenomenological and neurobiological perspective, the dialectic between similarity and otherness is manifested in embodied simulation, which is based on two simultaneous principles: first, similarity, whereby the other is implicitly perceived as an extension of the self; and second, distinction between self and other (29–32). The mirror neuron system is posited to underlie processes of similarity, which are analogous to the psychoanalytic concept of projective identification (33, 34). Conversely, multisensory integration, which is facilitated by the motor system and the experience of affective touch, is essential for perceiving the bodily self as distinct and separate from the other (35).

## Affiliative touch and psychosis

**Figure 1:**
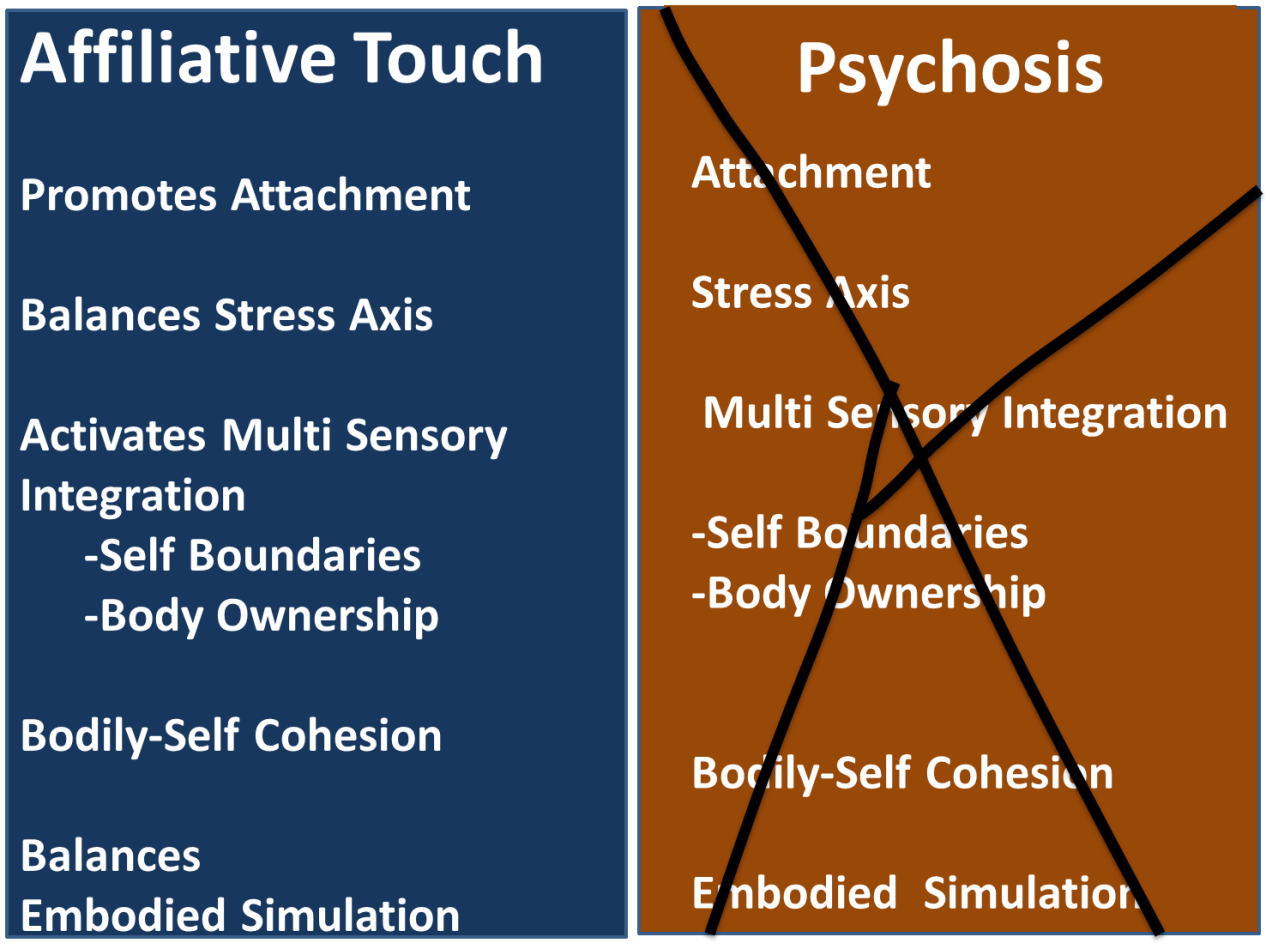
Affiliative touch functions altered in psychosis.

The various functions described, in which the affiliative touch is involved, are frequently impaired in individuals diagnosed with psychosis (**Figure 1**).

## Attachment disorders and traumatic alterations of the stress axis

A substantial body of evidence indicates a correlation between attachment disorders and the onset of psychosis in adults (36, 37).

Research suggests that early exposure to adverse childhood experiences, including neglect (characterized by a lack of emotional and physical care by the caregiver), sexual abuse and bullying, is associated with an increased risk of psychotic symptoms (38–41), by impairing the development of secure attachment (36, 37).This risk is associated with permanent alterations in the hypothalamicpituitary-adrenal (HPA) axis (42) and the impairment of oxytocin’s ability to regulate stress hormones such as cortisol (43).

The likelihood of developing psychosis in individuals who have experienced traumatic events during childhood is estimated to be three to fifteen times higher than in those who have not, depending on the nature and frequency of the trauma experienced (44–46).

## Psychotic alterations of the sense of self

### Alterations of the bodily self

From a phenomenological perspective, disturbances in the sense of self are underpinned by alterations in the bodily self. This is the primary feature of the psychopathology of schizophrenia (47, 48).

In the psychoanalytic field, disturbances in the sense of self have also been observed in patients diagnosed with schizophrenia (49, 50). It has been hypothesized that disturbances in self representation and self-awareness are the result of disturbances in multisensory integration (51).

Cognitive neuroscience has recently provided ample empirical evidence for alterations in the multisensory integration processes underlying bodily self-disorders, which would profoundly alter the complex structure of the self (32).

### Alterations of the boundaries of the self and ownership

The boundaries of the self are often disrupted in patients diagnosed with schizophrenia, as observed by pioneers of psychoanalytic treatment of psychosis such as Tausk (52) and Federn (24).

The neuroscientific literature has identified the significance of multisensory integration of disparate body signals in the formation of a sense of body ownership and the differentiation between one’s own body and that of others (53–56). This process involves the posterior cortex of the insula (57).

Individuals diagnosed with first-episode schizophrenia demonstrate alterations in the distinction between the self and the other, as well as changes in the activity of the posterior insula cortex, when observing stimulation based on affective touch. This form of touch integrates internal and external sensory signals and contributes to multisensory integration, the definition of self-boundaries, and the experience of ownership (20, 32).

Patients diagnosed with schizophrenia exhibit reduced interoceptive accuracy (58), a measure of body awareness and insula function (57). Furthermore, in patients diagnosed with schizophrenia, anatomical and functional changes have been observed in the insular cortex (59–61), a key region in the interoceptive processing of the sense of self that is directly linked to affective tactile C receptors (10–12).

### Embodied simulation disorders

Embodied simulation offers a basis for experimental examination of the balance between similarity and differentiation in intersubjectivity (29–32). As previously stated, the mirror neuron system plays a pivotal role in the perception of similarity (34). Conversely, multisensory integration, which is supported by the motor system and affective touch, is essential for the differentiation of the bodily self from others (35).

In recent years, dysfunctions of embodied simulation have been documented in patients diagnosed with schizophrenia. These studies have revealed impairments in mirror neuron maps, which influence the perception of similarity (62–65), and in sensorimotor circuits, which are associated with self/other differentiation (59, 61, 66, 67).

## Discussion

Affiliative touch has the potential to be an effective therapeutic intervention for psychosis for a number of reasons. Firstly, it has been shown to foster attachment and modulate the traumatic stress response (5–8). Secondly, it promotes multisensory integration, which contributes to the formation of the bodily self and a sense of ownership (18–20). Thirdly, it enhances the exchange of interoceptive and exteroceptive sensory information, which reinforces the boundaries of the self (8, 9). Finally, it stimulates the insula and interoception, facilitating bodily awareness (10–12). Moreover, the activation of both exteroceptive and interoceptive pathways by affiliative touch (8, 9) may facilitate a balanced relationship between otherness and similarity in embodied simulation, ultimately enhancing the quality of intersubjective relationships.

The therapeutic use of affiliative touch may be constrained by certain limitations, particularly in the case of patients who have experienced childhood trauma. A study conducted by Maier et al. (2020) (68) demonstrated that adults who have undergone childhood maltreatment are prone to experiencing discomfort in the presence of affiliative touch. It is therefore necessary to exercise caution in this regard.

The pharmacological use of oxytocin through exogenous intranasal administration acts on a broad range of unspecified brain areas, resulting in the inability to achieve the same therapeutic effects as affiliative touch. The latter produces elevated oxytocin levels at targeted nerve endings and receptors (69).

Given the documented effects of affiliative touch on various functions that are disrupted in individuals with psychotic disorders, there is a clear need for research to investigate the potential therapeutic benefits of affiliative touch. Despite this, and despite the considerable amount of basic research that has been conducted on affiliative touch, a recent systematic review (70) found that there has been minimal investigation into the clinical applications of affiliative touch in psychosis, with only a single study providing some evidence (71).

In this study, researchers developed a novel sensorimotor intervention for patients with psychosis, called Amniotic Therapy. This intervention integrates elements of affiliative touch and early parent-child interaction movements. The warm water environment of amniotic therapy facilitates the seamless combination of affective touch and movement (13, 72, 73). The results of this new treatment are promising, showing improvements in symptoms, social relationships and employment (72).

In one particular case, interoceptive accuracy was measured by observing an increase in its values after three years of therapy, indicative of an increase in interoception concomitant with a strengthening of the boundaries of the self (71, 73). However, the small sample size limits the generalizability of the results.

The objective of this paper is to stimulate further research and clinical applications of affective touch in psychosis, specifically in relation to the sense of self. Although this area is under-researched, it has significant therapeutic potential without harmful side effects. This contribution to the discussion of affective touch and its relationship to the sense of self aims to stimulate further research and encourage its clinical application.
